# Adapted Deep Ensemble Learning-Based Voting Classifier for Osteosarcoma Cancer Classification

**DOI:** 10.3390/diagnostics13193155

**Published:** 2023-10-09

**Authors:** Md. Abul Ala Walid, Swarnali Mollick, Pintu Chandra Shill, Mrinal Kanti Baowaly, Md. Rabiul Islam, Md. Martuza Ahamad, Manal A. Othman, Md Abdus Samad

**Affiliations:** 1 Department of Computer Science and Engineering, Khulna University of Engineering and Technology, Khulna 9203, Bangladesh; abulalawalid@gmail.com (M.A.A.W.);; 2 Department of Computer Science and Engineering, Northern University of Business and Technology, Khulna 9100, Bangladesh; 3 Department of Computer Science and Engineering, Bangabandhu Sheikh Mujibur Rahman Science and Technology University, Gopalganj 8100, Bangladesh; baowaly@bsmrstu.edu.bd (M.K.B.);; 4 Department of Biomedical Engineering, Islamic University, Kushtia 7003, Bangladesh; 5 Medical Education Department, College of Medicine, Princess Nourah bint Abdulrahman University, Riyadh 11671, Saudi Arabia; maothman@pnu.edu.sa; 6 Department of Information and Communication Engineering, Yeungnam University, Gyeongsan-si 38541, Republic of Korea

**Keywords:** bone malignancy, convolution neural network (CNN), histopathological image classification, osteosarcoma, transfer learning, ensemble learning

## Abstract

The study utilizes osteosarcoma hematoxylin and the Eosin-stained image dataset, which is unevenly dispersed, and it raises concerns about the potential impact on the overall performance and reliability of any analyses or models derived from the dataset. In this study, a deep-learning-based convolution neural network (CNN) and adapted heterogeneous ensemble-learning-based voting classifier have been proposed to classify osteosarcoma. The proposed methods can also resolve the issue and develop unbiased learning models by introducing an evenly distributed training dataset. Data augmentation is employed to boost the generalization abilities. Six different pre-trained CNN models, namely MobileNetV1, Mo-bileNetV2, ResNetV250, InceptionV2, EfficientNetV2B0, and NasNetMobile, are applied and evaluated in frozen and fine-tuned-based phases. In addition, a novel CNN model and adapted heterogeneous ensemble-learning-based voting classifier developed from the proposed CNN model, fine-tuned NasNetMobile model, and fine-tuned Efficient-NetV2B0 model are also introduced to classify osteosarcoma. The proposed CNN model outperforms other pre-trained models. The Kappa score obtained from the proposed CNN model is 93.09%. Notably, the proposed voting classifier attains the highest Kappa score of 96.50% and outperforms all other models. The findings of this study have practical implications in telemedicine, mobile healthcare systems, and as a supportive tool for medical professionals.

## 1. Introduction

Less than 0.2% of all cancer cases are predominant bone cancers, which are exceptionally infrequent tumors whose true incidence is difficult to ascertain due to their rarity [1]. The three predominant forms of bone cancer are osteosarcoma, chondrosarcoma, and Ewing sarcoma. The histological lineage of different bone cancer types determines their nomenclature. Osteosarcomas arise from bone tissue; a chordoma originates from notochordal tissue; and chondrosarcomas emerge from cartilage tissue. Primary bone cancers exhibit significant clinical variability and are frequently curable when given appropriate care. The incidence of bone cancers exhibits variations in both sex and age. With the highest prevalence in the fifth to sixth decades of life, chordoma is more prevalent in men. Adults who are middle-aged or older are chondrosarcoma carriers, and younger generations and children are barriers to Ewing sarcoma and osteosarcoma. The tumor leads to significant skeletal transformation, cracks, distress, and malnutrition once it has spread to the bone, making it a leading cause of mortality and morbidity. Patients diagnosed with advanced breast, prostate, and lung cancer often encounter bone cancer discomfort due to the notable tendency of these malignancies to metastasize to the skeletal system [2]. Osteosarcoma stands in the eighth position among all cancers in children. It usually starts in the bone cells, forming new bone tissue, and can develop in any bone in the body. However, it most commonly occurs in the long legs and arms. The percentage of the most frequent sites of osteosarcoma is 42% for the femur, 19% for the tibia, and 10% for the humerus [3]. The 10- to 14-year-olds experience the first peak, and adults over 65 experience the second. Per year, 3 million people are affected by osteosarcoma. However, the age group of 15 to 19 is more affected by the health problem. In general, the incidence rate of females is lower than that of males [4].

Symptoms of osteosarcoma can include pain, swelling, stiffness in the affected bone, and difficulty moving the affected limb. A mass or lump may be visible on or near the affected bone. The etiology of osteosarcoma remains uncertain, although certain risk factors have been identified, including a prior history of radiation therapy, the presence of specific genetic disorders such as Li-Fraumeni syndrome, and a previous diagnosis of Paget’s disease. Spinal osteosarcoma is an aggressive form of bone cancer primarily affecting the spine. Compared to osteosarcoma of the extremities, which has a mean age of 38, osteosarcoma of the spine typically affects older age groups [5]. The danger lies in its ability to rapidly grow and spread (metastasize) to other body parts, including the lungs. Due to its location near critical nerves and the spinal cord, it can cause severe pain, neurological deficits, and even paralysis. osteosarcoma has a significantly greater death rate than other cancers. Early identification is crucial in these circumstances since it may lower the death rate. Crucial diagnostic tools for osteosarcoma include magnetic resonance imaging, X-rays, and histological biopsy tests. Presently, thorough clinical records are taken at the introductory level of osteosarcoma diagnostic tests and physical exams [6]. To diagnose osteosarcoma, the knowledge level and experience of the doctor should be proper and high. It can be challenging to distinguish the subtleties of histological images because pathologists must look at many histological slides [7]. In this context, the use of an automated method for osteosarcoma detection has the potential to alleviate the burdens and obligations faced by pathologists due to the overwhelming volume of cases.

Furthermore, numerous laboratory tests are required due to the rising incidence of cancer, which frequently causes pathologists to become exhausted. Cancer management and diagnostic tests are currently more complicated than ever due to patient-specific treatments [8]. In recent years, there has been a notable rise in the utilization of automated analysis techniques for microscopic image examination in the context of cancer detection. This trend has emerged as a response to the limitations posed by conventional methods. Radiologists and pathologists can use computer-aided detection (CAD) technology to immediately find neoplasms depending on histopathology image data [9,10]. Histological slides are now being converted into digital image datasets in a trend that enables machine learning (ML) to cooperate on photographic files to improve accurate diagnosis. CAD innovation that incorporates potent algorithms, like deep learning (DL) models, which can precisely identify cancerous tumor growth. Researchers have conducted several clinical studies on various illnesses, including osteosarcoma. ML is very efficient for processing digital images and can easily detect and classify osteosarcoma. In the detection of osteosarcoma, researchers have utilized ML and DL approaches, such as convolutional neural networks (CNNs), Support Vector Machines (SVMs), and several other strategies [11]. The CNN model with data augmentation was employed by Asmaria et al. [12] as one of the strategies to enhance the performance of the model.They used MATLAB to build the CNN model. Their model performs well in classifying osteosarcoma, and the accuracy reaches 95.37%. Mahore et al. [13] employed various ML algorithms, including Decision Tree (DT), Support Vector Machine (SVM), K-Nearest Neighbors (KNN), and AdaBoost (Adaptive Boosting), to conduct a comparative analysis of the classification of osteosarcoma. The findings revealed that AdaBoost outperformed the other algorithms, achieving an accuracy rate of 91.70%. Several studies have demonstrated the reliable prediction of osteosarcoma using DL systems. The goals of the proposed work are to ensure the development of an expert system to diagnose osteosarcoma, which will aid doctors in treating patients more quickly and effectively, to provide the proposed system as telemedicine since sophisticated diagnostic equipment is not readily available in most rural areas, and to use the proposed system as a smart hospital management system in diagnostic centers.

This study presents evidence of the efficacy of DL-based tools in accurately detecting osteosarcoma tumors. The study utilizes a publicly available dataset and employs a sophisticated classification system incorporating a proposed CNN architecture and a CNN-based voting classifier. This approach, known as heterogeneous ensemble learning (ENL), aims to ensure appropriate patient treatment. The fundamental principle behind ENL resides in amalgamating the predictions derived from multiple models, potentially yielding superior outcomes compared to utilizing any singular model in isolation [14]. The proposed voting approach’s concepts enhance the majority voting strategy [15], meticulously designed to address and rectify significant limitations. The dataset of pathology archives from the Children’s Medical Center [16] has been processed to DL algorithms to facilitate subsequent research to classify tumor, non-tumor, and necrotic tumor cells. Our dataset has uneven distribution, which may cause the splitting strategy to accept an imbalance landmark in the training set. For the unevenly distributed dataset, the biases exhibited by the models may stem from a tendency to prefer a group with a larger population [17].

Bias in ML is widely regarded as a problematic factor [18]. Our solution introduces a way for lowering biases to generate a DL model free of any slant. Six modified transfer learning approaches, namely MobileNetV1 [19], MobileNetV2 [20], Res-NetV250 [21], InceptionV2 [22], NasNetMobile [23], and EfficientNetV2-B0 [24] are treated. The improved performance of the adapted transfer learning model over its predecessor architecture can be seen in each scenario. The upper layer has undergone adjustment to optimize the product. Frozen and fine-tuned-based phases are applied to train and assess six distinct transfer learning models. A CNN model with a custom-built architecture is also designed and developed by adapting and enhancing the concept outlined in [25] to classify osteosarcoma. A comparative analysis has been made. The suggested CNN architecture trained with a balanced training set achieves an accuracy of 95.63%. It outperforms ordinary and fine-tune-based pre-trained models developed from balanced and imbalanced training sets. Moreover, the ENL-based proposed max voting classifier prepared from the proposed CNN, fine-tune-based NasNetMobile, and EfficientNetV2B0 base learner, designated as ENL-CNE, has achieved 96.51% accuracy and outperforms all other models. For the group of cancerous tumors, the proposed ENL model achieves the highest recall, which equals 100%. The subsequent section analyzes the contributions of this study.

- A structured dataset for ML-based osteosarcoma classification was constructed. An augmentation strategy into the training data was incorporated.
- In transfer learning, six pre-trained CNN models were applied to the dataset for classifying osteosarcoma. An optimal pre-trained model using fine-tuning by unfreezing the entire model was developed.
- A CNN architecture was developed that, with a balanced dataset, makes classification more effective and gives a faster classification rate.
- An adapted heterogeneous ENL-based voting classifier and brute-force strategy were constructed to evaluate all combinations of base learners systematically.
- The performance of all the learning models used in this study and comparisons among them were analyzed.

The remainder of this study is structured as follows. In Section 2, the literature review has been covered. In Section 3, the research technique is presented. Details of the implementation are presented in Section 4. The result analysis is shown in Section 5. Finally, Section 6 summarizes the results and discusses potential future studies.

## 2. Literature Review

The following discussion draws on various available literature concerning the diagnosis of osteosarcoma. Ahmed et al. [26] proposed a compact CNN architecture to classify small and imbalanced osteosarcoma histology image datasets. The study employed an over-sampling technique to mitigate class imbalance and overfitting. Experimental results demonstrate that the proposed CNN models achieve high accuracies, with the non-regularized model attaining 78% testing accuracy for the imbalanced dataset and 81% testing accuracy for the balanced dataset. The regularized model achieves 75% testing accuracy for the imbalanced dataset and 86% testing accuracy for the balanced dataset. Ahmed et al. [26], Gawade et al. [27], Vezakis et al. [28], and even our study utilizes a similar dataset for the analysis. The dataset employed in these studies consists of microanatomy images of hematoxylin and Eosin-stained osteosarcoma collected by a group of clinical professionals from the University of Texas at Dallas.

Gawade et al. [27] proposed an automatic DL approach for detecting osteosarcoma bone cancer using CNN-based models. The researchers examined four algorithms to construct their conceptual framework: VGG16, VGG19, DenseNet201, and ResNet101. In their study, the authors [27] used various performance metrics to assess the effectiveness of their approach. The study used performance metrics, including accuracy, F1 score, precision, recall, AUC, and Vscore, to evaluate the performance. The findings indicated that the ResNet101 model exhibited superior performance compared to the other models, attaining the greatest accuracy rate of 90.36%, F1 score of 89.35%, precision of 89.51%, recall of 89.59%, AUC of 0.946, and Vscore of 2.720.

Furthermore, Vezakis et al. [28] intended to demonstrate the efficiency of 12 pre-trained DL models for osteosarcoma classification, emphasizing the importance of selecting models with smaller parameter sizes. They split the dataset into 70% for training and 30% for testing. The pre-trained models were fine-tuned using the PyTorch framework, and the top-performing networks with the appropriate image input size were selected. On average, MobileNetV2 was identified as the best-performing model based on the macro-average F1 score.

However, Shen et al. [29] are devoted to the field of ML and conducted a study to classify osteosarcoma and benign tumor patients using ML algorithms, specifically Random Forest (RF) and Support Vector Machine (SVM). They utilized image features and metabolomic data, evaluating model performance based on accuracy, sensitivity, specificity, P-value, and AUC. The study involved X-ray image segmentation, feature extraction, selection, and ML-based categorization. To increase the accuracy of the models, they used 5-fold cross-validation. The RF model achieved an accuracy of 85%, sensitivity of 92%, specificity of 78%, *p*-value of 0.044, and AUC of 0.94. In contrast, the SVM model achieved an accuracy of 81%, sensitivity of 81%, specificity of 80%, *p*-value of 0.080, and AUC of 0.86. The performance analysis demonstrates that the RF model outperformed the SVM model. On the other hand, Nabid et al. [30] introduced a sequential Recurrent Convolutional Neural Network (RCNN) comprising CNN and bidirectional Gated Recurrent Units (GRU) for osteosarcoma classification. The model’s performance was enhanced using strain normalization techniques. Using the osteosarcoma histopathological image dataset, a comparison was made with the pre-trained models, including AlexNet, ResNet50, VGG16, LeNet, and SVM. In [30], a method was proposed consisting of four Histology Region Convolution (HRC) blocks, followed by bidirectional Gated Recurrent Units (GRU) and dense networks. It achieved an accuracy of 89%, precision of 88%, recall of 89%, and F1 score of 89%. The area under the ROC curve for non-tumor, viable tumor, and necrotic cells were 0.9, 0.86, and 0.88, respectively.

Anisuzzaman et al. [31] investigated the effectiveness of DL-based pre-trained models for osteosarcoma detection using a public histological image dataset. The objective was to distinguish necrotic images from non-necrotic and healthy tissues. The novelty of the proposed approach in [31] lies in applying pre-trained models to different dataset categories, using the entire tile image as input. Without patches, transfer learning techniques such as InceptionV3 and VGG19 were utilized on Whole Slide Images (WSI). Both binary and multi-class classification were performed using VGG19 and InceptionV3. The models were trained for 1500 epochs with an Adam optimizer and a learning rate of 0.01. The VGG19 model demonstrated the best level of accuracy across all scenarios. In addition, Mishra et al. [32] proposed using CNN to enhance the efficiency and accuracy of classifying osteosarcoma tumors into tumor classes (viable tumor, necrosis) versus non-tumor. Their study introduces a novel application of CNN designed for osteosarcoma image classification. The dataset employed in their study comprised one thousand images categorized as Viable, Necrosis, and Non-Tumor.

On the contrary, certain investigations are undertaken utilizing genome data. To examine the expression profile of repetitive elements (RE) in osteosarcoma, Ho et al. [33] conducted their study. They analyzed the entire RNA of 36 fresh-frozen paired samples from osteosarcoma patients, 18 of which were tumors and 18 of which were not. They discovered that Eighty-two repetitive DNA elements (REs) expressed differentially in osteosarcoma and normal bone. A total of 35 REs were up-regulated, and 47 were down-regulated out of all the significantly altered REs. Reimann et al. [34] identify innovative biomarkers for osteosarcoma. The genes in which the mutations were identified can be regarded as potential candidates for the identification of biomarkers for osteosarcoma. In the exome of the tumor, a comprehensive analysis revealed extensive genomic rearrangements that meet the criteria for chromotripsis. Next-generation sequencing was employed to analyze the complete exome of both tumorous and non-tumorous bone tissue samples obtained from a patient diagnosed with osteosarcoma. Multiple software programs were used for data processing, in which exome data were integrated with RNA-seq data. Their investigation identified about three thousand somatic single nucleotide variations (SNVs) and minor insertions or deletions, as well as over two thousand copy number variants (CNVs) distributed across various chromosomes. They also observed that somatic modifications are specifically related to the development of bone tumors, while germline mutations are related to the occurrence of cancer in a broader sense.

The work introduces a CNN architecture consisting of three sets of convolutional layers paired with corresponding max-pooling layers, which are employed to enhance the feature extraction process. Additionally, two fully connected layers are used to enhance data augmentation. The researchers explored different baseline architectures with varying hidden layers to optimize performance. The extended neural network version (with increased hidden layers and decreased filter size from 5 × 5 to 3 × 3) outperformed the simple baseline architecture. The accuracy rates for different classes in the baseline implementation and the proposed architecture were as follows: Viable—83% and 92%, Necrosis—73% and 90%, and Non-Tumor—91% and 95%, respectively. Moreover, the average accuracies of AlexNet, LeNet, VGGNet, baseline architecture, and their proposed architecture were 73%, 67%, 67%, 84%, and 92.40%, respectively. Asito et al. [35] proposed a computer-aided diagnosis system using CNNs for osteosarcoma detection on bone radiography. They employed a window-based approach, where CNNs were applied to classify each window and identify cancer-affected regions in the image. The dataset used in the study originated from a study conducted at the University of Sao Paulo. The windows were categorized as normal or tumor (osteosarcoma) using CNNs, comparing their custom CNN model and a pre-trained VGG16. Beyond these techniques, Decision Tree, Random Forest, MLP, and MLP with feature selection classifiers were employed. The pre-trained CNN achieved the highest accuracy of 77% and the highest sensitivity of 84%, and the MLP with feature selection algorithm also achieved the highest sensitivity of 84%. The MLP attained the highest specificity of 76%. These findings highlight the effectiveness of CNNs in osteosarcoma detection on bone radiography and demonstrate the superior performance of the pre-trained VGG16 compared to the other models.

## 3. Research Methodology

**Figure 1:**
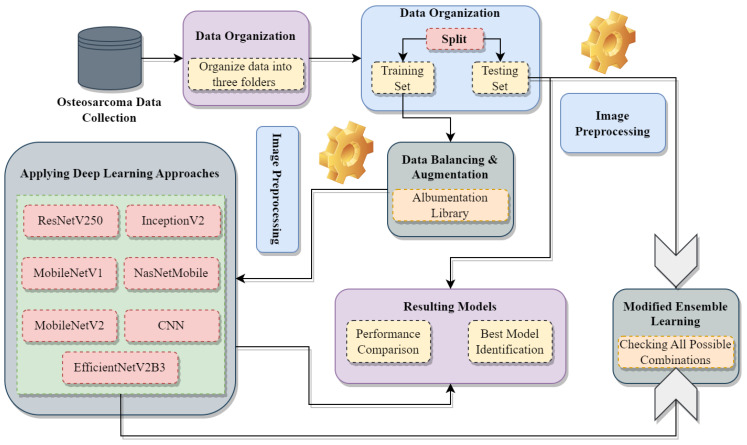
Proposed methodology.

In this section, the research methods used for the study have been illustrated. Figure 1 concisely demonstrates the proposed methodology. The following phases are used to develop our study. After obtaining the dataset from the Cancer Imaging Archive, the dataset is organized into three folders and known as class names. Next, the dataset is divided into two portions: 80% for training and 20% for testing. The raw dataset is highly imbalanced, so data balancing has been performed on the training set using a data augmentation library named “Albumentations”. The minority classes have been over-sampled to the highest class. Subsequently, the training and test sets have undergone image preprocessing procedures, including image normalization.

**Figure 2:**
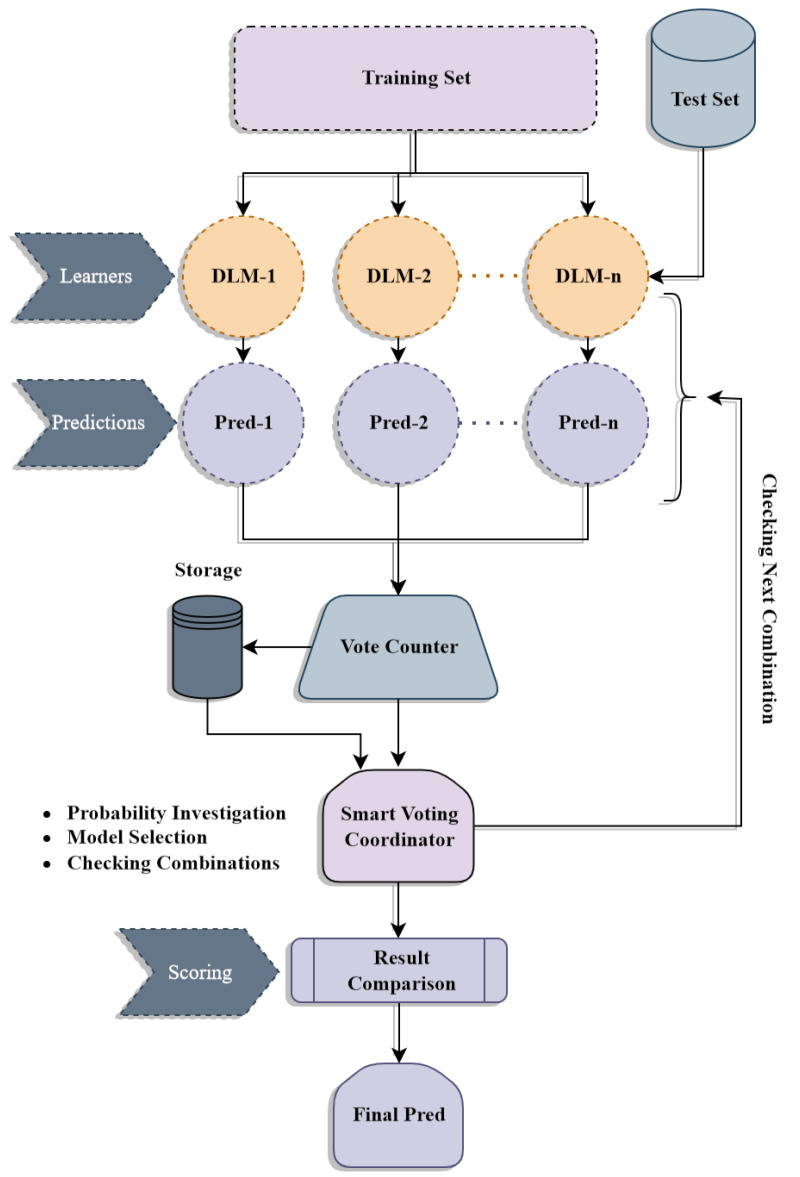
Functioning of the proposed majority voting ensemble algorithm.

A CNN model with a customized architecture tailored for this study undertaking and six other deep transfer learning pre-trained CNN models, namely Mo-bileNetV1, MobileNetV2, ResNetV250, InceptionV2, NasNetMobile, and EfficientNetV2B0 have been applied to the training set. Every model has undergone a comprehensive evaluation, culminating in a comprehensive examination of the collective findings. Additionally, an adapted voting classifier is shown in Figure 2, which constitutes a specialized implementation of heterogeneous ENL, has been devised, and certain drawbacks are also mitigated.

The ENL approach is heterogeneous, as the constituent base models encompass diverse types [36]. Adopting the max voting technique is intended to improve the effectiveness of DL classifiers [37]. Algorithm 1 demonstrates the proposed modified majority voting ensemble approach. In this approach, the vote counter tallies the votes from various algorithms for each category corresponding to every testing instance and stores them in CF. Subsequently, the final prediction FPrei describes the category that garners the highest frequency value. The drawbacks, like two or more categories occurring the same number of times, are addressed by incorporating class probability, as outlined in lines 16–21 of Algorithm 1. As depicted in Figure 2, the smart voting coordinator effectively overcomes these limitations by deriving the ultimate output from the highest frequency value obtained through the vote accumulation facilitated by the vote counter. Subsequently, the smart voting coordinator utilizes a brute-force mechanism to assess every conceivable combination of the underlying base learners rigorously. Wherein a combination comprises a minimal count of base learners, precisely two. Such strategic coordination ensures a robust and accurate final prediction. Reduced mortality upon osteosarcoma diagnosis is the main objective in clinical procedures. The early-stage tumor must be kept from metastasizing at all costs. In addition to lowering the likelihood of a false positive, early automatic detection can also be used to support the physician in deciding whether metastasis has occurred. Using CNN, computer-aided technology, the effort of the physician can be significantly reduced, and patient outcomes can be improved. Algorithm 1 describes the DL models used in this study. **Algorithm 1** Adapted Majority Voting Ensemble Algorithm
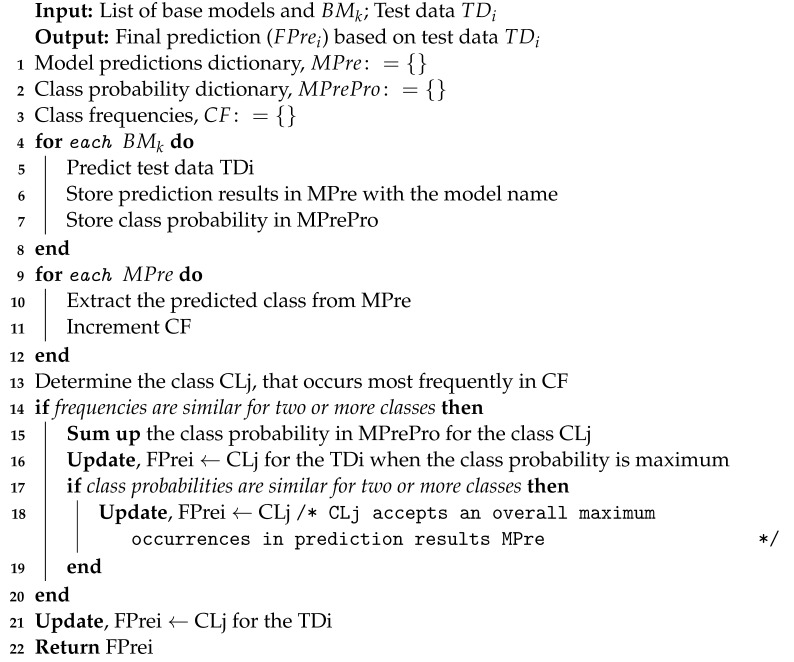


### 3.1. Deep Learning Algorithms

This section will comprehensively discuss the deep learning (DL) methods employed in our investigation. The fundamental elements of the deep CNN model, together with six additional pre-trained deep transfer learning models, namely MobileNetV1, MobileNetV2, ResNetV250, InceptionV2, NasNetMobile, and EfficientNetV2B0, have been elucidated.

1. **CNN:** Among all DL networks, CNN is widely utilized, particularly for computer vision activities. Soon afterward, in Waibel et al. [38] and Lecun et al. [39] developed two different architectures of CNNs for phoneme recognition that shared weights between temporal receptive fields and back-propagation training and a useful CNN architecture for document recognition, respectively. CNN belongs to DL networks and is a supervised ML algorithm. The key convenience of CNN is that it can automatically extract essentials from the dataset compared to its predecessors [40] as it consists of some primary layers [41]. The subsequent section delineates its several layers.
  - Convolutional Layers: It is one of the most significant layers of CNN. In this layer, kernels or filters of weights are convoluted for feature extraction, which is the main benefit of CNNs.
  - Pooling Layers: The main objective of the pooling layer is to decrease the spatial dimensions of the input image systematically, therefore reducing the computational load imposed on the network. In CNN, pooling reduces the size of the down-sampling operation. It sends only the most crucial data to subsequent layers.
  - Dropout Layers: The dropout layer drops random nodes to reduce overfitting. The main goal of the dropout layer is to drop random nodes throughout various iterations of the process and introduce variability and non-linear effects to the training set [42].
  - Fully Connected Layers: The fully connected layer is one of the most elemental components in CNN. The final several layers of the network are known as fully connected layers. The fully connected layer is responsible for receiving the output from the preceding pooling or convolutional layer. Prior to its application, the output is flattened. In a fully connected layer, the input first undergoes multiplication by a weight matrix and then an addition of a bias vector [43].
2. **MobileNetV1:** MobileNet is a pre-trained model in Transfer Learning of CNN architecture, trained with the ImageNet dataset. Its creation aimed to optimize precision, considering the constraints imposed by the restricted resources typically available for on-device or embedded applications. The foundation of MobileNet is depthwise separable convolutions, which have pointwise and depthwise convolutions as their two main internal layers. Filtering the input without adding new features is called depthwise convolution [44]. Thus, pointwise convolution—a technique for creating additional features—was merged. Depthwise separable convolution is the name given to the two layers together. Each input channel underwent a singular filter application through depthwise convolutions. The resulting output from the depthwise layer was subsequently merged in a linear manner using 1 × 1 convolutions (pointwise).Following each convolution, the techniques of batch normalization (BN) and rectified linear unit (ReLU) were applied [45].
3. **MobileNetV2:** MobileNet network is frequently a pre-trained model in CNN architecture’s Transfer Learning, trained on the ImageNet dataset. With 1.4 million photos and 1000 classes of online images, the ImageNet dataset was used as MobileNetV2’s pre-trained training set. MobileNetV2 is a lightweight neural network. MobileNetV2’s fundamental architecture is based on that of MobileNetV1, its predecessor. Fifty-three layers make up the CNN known as MobileNetV2. Google Inc. has published MobileNetV2 [46]. MobileNetV2 employs linear bottlenecks to implement the depthwise separable convolutions (DSC) technique for probabilistic computations. Such a technique focuses on the problem of information degradation within non-linear layers seen in convolutional blocks.It is a very efficient feature extractor for image classification [47].
4. **ResNetV250:** In 2016, He et al. [48] developed a deep residual network or ResNet model. ResNet network is a pre-trained model in Transfer Learning of CNN architecture, trained with the ImageNet dataset. DL training has several challenges, including time consumption and limited layers. The study [49] was created to address the complexity of DL training. The computation time of ResNet has made it more efficient; it takes low computation time, and the ability to train is excellent. Vanishing gradient and K. He degradation problems are there in deeper neural training. When ResNet has 50 layers total, then it is called ResNet50. The residual network architecture’s capacity to accept images of sizes different from those used for training is another reason to use it. The ImageNet dataset is responsible for the weights used in ResNet.
5. **InceptionV2:** The inception-v2 network is frequently pre-trained in CNN architecture’s Transfer Learning. It is the second generation of the inception convolutional network. Batch normalization is prominently used in Inception-v2. In addition, dropout and local response normalization have been eliminated due to the advantages of batch normalizing. It takes 224 × 224 sized images as its input. The architecture of inception-v2 includes 3 × 3 sized filters, whereas inception-v1 has 5 × 5 sized filters, making the second inception version faster [50].
6. **NasNetMobile:** The NasNetMobile is a CNN trained on a dataset consisting of more than one million images obtained from the ImageNet collection. The Neural Architecture Search Network was conceived and developed by the Google Brain team. It is an adaptable CNN architecture where reinforcement learning is used to optimize the building blocks (cells). It comprises normal and reduction cells, its two primary functionalities [51]. NasNet designs come in two major varieties: NASNetLarge and NasNetMobile. According to the network’s necessary capacity, a cell comprises just a few processes and is repeated several times.
7. **EfficientNetV2B0:** An efficient network is a pre-trained model in the CNN architecture’s Transfer Learning that was trained using the ImageNet dataset. The efficient network initially proposed by Tan and Le Deng et al. [52] was termed EfficientNet. The EfficientNet model has eight varieties. The EfficientNet series network can be subdivided into eight sub-networks, B0–B7, based on the degree of the scale, with each model number corresponding to a version with more parameters and greater accuracy. Google AI created the model, and it is accessible through GitHub repositories. Transfer learning is used in the EfficientNet architecture to save processing time and power. The EfficientNet Models have scaled CNN models that have already been trained and may be applied for transfer learning in image classification issues [53]. Tan and Le [54] further enhanced the Efficient network in 2021, named the EfficientNet-V2 network. They divided the enhanced Efficient network into S, M, and L sub-networks. After experimental validation, the new network is more efficient, consumes fewer resources, and has greater real test accuracy than the previous EfficientNetV1 [54].

### 3.2. Data Collection

**Figure 3:**
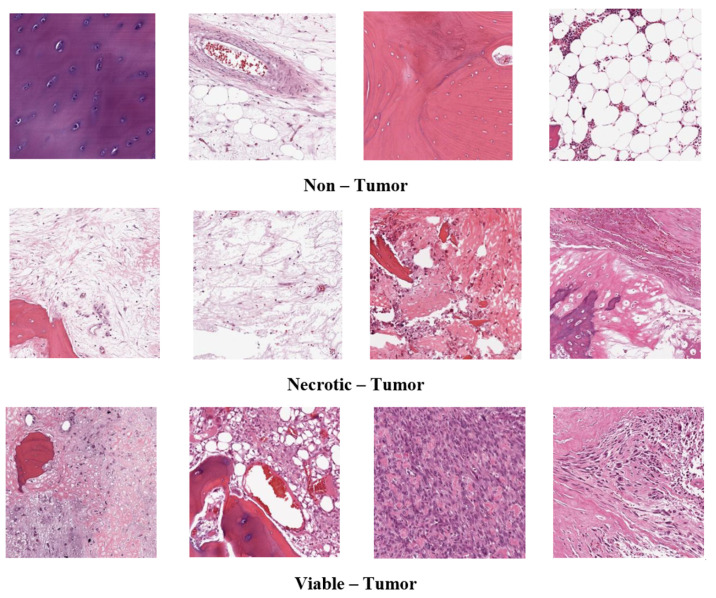
Example images of osteosarcoma histology image dataset at 10× magnification.

The dataset for this investigation was obtained from the Cancer Imaging Archive website [11]. The dataset named “Osteosarcoma Data from UT Southwestern/UT Dallas for Viable and Necrotic-Tumor Assessment (Osteosarcoma-Tumor-Assessment)” contains 1144 images of size 1024 × 1024 at 10× resolution. It consists of histology images of osteosarcoma stained with hematoxylin and eosin (H&E). The histology images included in the dataset were obtained from Children’s Medical Center, Dallas. The dataset encompasses a total of 50 patients who were treated at the medical center throughout the period spanning from 1995 to 2015. The images in Figure 3 are categorized based on the predominant type of cancer present. These categories include Non-Tumor, which indicates the absence of tumor cells; Viable Tumor, which indicates the presence of actively growing tumor cells; and Necrosis Tumor, which indicates the presence of tumor cells that have been destroyed. Among these, the non-tumor category comprises a total of 536 histological photographs. The viable-tumor category encompasses 345 images, while the necrotic-tumor category includes just 263 histological images.

### 3.3. Data Preprocessing and Normalization

Image preprocessing is a technique employed to prepare images for utilization in model training and inference. Additional preprocessing processes encompass resizing, orienting, and color modifications. Preprocessing aims to improve picture data that reduces unintentional distortions or increases visual properties crucial for further processing. The size of the images within the dataset utilized in the present investigation is 1024 by 1024 pixels. The images were resized into 224 × 224 pixels to make the computations faster. Normalization is a technique used in image processing to modify the range of pixel luminance levels. The typical function of image normalization is to transform an input image into pixel levels that are more conventional or comfortable to the senses. The images consist solely of a composite of distinct pixel values dispersed across the range of 0 to 255. Working with huge values is impractical and time-consuming, necessitating more capable computing devices. However, the normalization process involves dividing the pictures by a value of 255, which reduces such burden.

### 3.4. Dataset Splitting

The dataset must be divided into a particular size for training and testing. We should keep most of the data from the training set rather than the testing set to build an accurate model [55]. In this study, the dataset was divided into 80% and 20% ratios for training and testing, respectively. A total of 10% of the training set examples were used as a validation set.

### 3.5. Dataset Balancing and Augmentation

**Figure 4:**
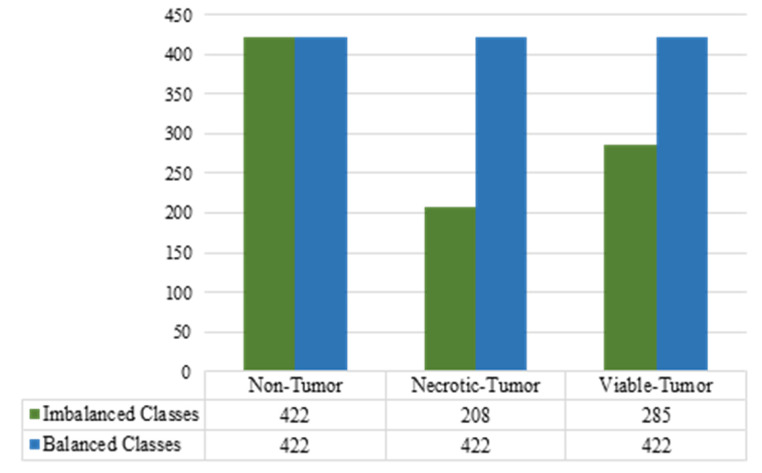
Data distribution of each class before and after balancing.

The dataset utilized in this study presents a highly imbalanced distribution, which significantly impacts the obtained results. Such data imbalance poses a considerable challenge, as it may introduce biases and hinder the effective application of traditional learning algorithms in real-world domains. A pivotal step has been taken to balance the dataset after splitting it into training, testing, and validation sets [56] with one of the data augmentations libraries named “Albumentations” [57]. Albumentations is a quick and adaptable open-source library for image augmentation that offers a wide range of image transform operations and functions as an intuitive wrapper for other augmentation tools [58]. After splitting the dataset, the training set contains the following images: the non-tumor class contains 422 images, the necrotic-tumor class contains 208 images, and the viable class contains 285 images. The minority classes in the training set have been over-sampled to the highest class. The number of necrotic-tumor and viable-tumor images have been over-sampled into 422 images. The training set was over-sampled using the technique of horizontal flipping. Figure 4 demonstrates the data distribution of each class before and after balancing. Our training dataset applies augmentation techniques such as vertical flip, rotation, and brightness adjustments. Data augmentation is a strategy employed to expand the volume of data utilized to train a model. DL models sometimes require significant training data to provide reliable predictions, which may not always be readily available. Consequently, the available data are expanded to enhance the development of a more comprehensive model. The ImageDataGenerator class from Keras API was used to ensure that the model is exposed to novel modifications of the images throughout each epoch. One notable benefit of utilizing the ImageDataGenerator is its ability to minimize memory use effectively.

## 4. Implementation Details

In this study, six transfer learning techniques, pre-trained CNN models, namely MobileNetV1, MobileNetV2, ResNetV250, InceptionV2, NasNetMo-bile, EfficientNetV2B0, applied in both frozen-based and fine-tuning phases with full model unfreezing, and a self-constructed architecture for the CNN model and innovative ENL approach, fortified by a brute-force mechanism, has been formulated to overcome the classification tasks with high exactitude.

### 4.1. Setup of Proposed CNN

**Figure 5:**
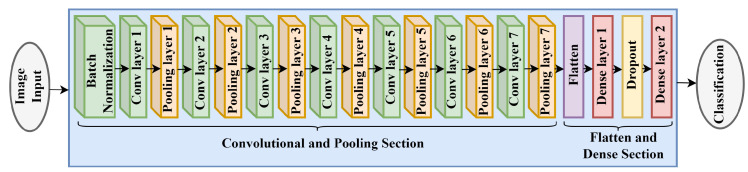
The architecture of the proposed CNN model.

Figure 5 shows the proposed architecture of the CNN model. In the proposed architecture, the convolution layer and pooling layers work simultaneously. First, batch normalization has been applied to normalize the input data and to make the model faster. The input and output size of the image array is the same as 224 × 224 × 3. Then, the input array size of the first convolution layer is 224 × 224 × 3 and generates 32 feature maps with the filter size 3 × 3. The max-pooling layer takes these feature maps and down-sample them into 112 × 112 × 3. The second convolution layer takes 32 inputs and makes it into 32 again. The second max-pooling layer reduces the size of the feature map from 112 × 112 to 56 × 56. The third convolution layer takes 32 inputs and makes it into 64 feature maps. The third max-pooling layer, along with certain crucial features, reduces the size from 56 × 56 to 28 × 28. The fourth convolution layer takes 64 inputs, and the feature maps remain 64. The fourth pooling layer decreases the size from 28 × 28 to 14 × 14 with some crucial features. The fifth convolution layer takes 64 inputs and makes it into 128 feature maps, and the fifth pooling layer reduces the size from 14 × 14 to 7 × 7. Finally, the sixth convolution layer takes 128 inputs. It makes it into 128 feature maps, and the pooling layer reduces the size from 7 × 7 to 3 × 3. Next, the seventh convolution layer takes 128 inputs. It makes it into 256 feature maps, and the pooling layer reduces the size from 3 × 3 to 1 × 1. The flattened layer converts the output shape 1 × 1 into 256 single nodes and passes to the first dense layer. The output shape of the first dense layer is 512 with the ReLU activation function and calculates 131,584 parameters. Then, one dropout layer was applied. The second dense layer contains three nodes of three classes with SoftMax activation function and calculates 1539 parameters. The total parameters of the architecture is 715,311. Table 1 demonstrates the layers of the proposed CNN model.

One of the numerous histological patterns that pathologists have linked to the disease, such as the osteoblastic, chondroblastic, or fibroblastic pattern, may have an impact on the diagnosis of osteosarcoma. CNNs distinguish between non-tumor and tumor tissues based on the inherent differences in pixel intensity patterns and spatial features within medical images [59], such as histological images, used in this study. CNNs leverage their ability to automatically learn and extract distinctive features from the images during training. For instance, tumors often exhibit irregular shapes, abnormal textures, or enhanced regions compared to surrounding healthy tissues. These unique characteristics, combined with the network’s learned filters, enable CNNs to identify subtle structural variations and intensity differences within the images [60]. Through a process of feature extraction and hierarchical representation learning, CNNs can effectively classify tissues as non-tumors or tumors in medical diagnosis and treatment planning.

### 4.2. Transfer Learning

A stored architecture that has already undergone training on a sizable dataset—typically an extensive image classifying task—is referred to as a pre-trained model. Transfer learning is an ML technique in which a model constructed for one task is utilized as the foundation for a model for another. The model construction can be formed by applying transfer learning to adapt the pre-trained network to a specific proposal or by utilizing the model in its standard form. It is the enhancement of learning by transferring information to a new task. The complete training process of a novel DL model might incur significant computing expenses. Furthermore, more datasets are needed for DL than conventional ML techniques. However, its progress is frequently constrained by the scarcity of histology and radiological images. These shortcomings are what transfer learning is meant to address [61]. The fundamental tenet of ML and DL algorithms is that training and prospect data should always be distributed across the same area. Difficulties arise in ML when insufficient training data are available for the given research topic. Consequently, the DL model can be taught using previously learned networks to derive the fundamental parameters, which can be applied to data sets from different areas. Learning outcomes can be improved if knowledge transmission is carried out effectively in these circumstances and limiting costly data labeling efforts.

### 4.3. Parameters Setup

There are different parameters used in proposed CNN models. Table 2 demonstrates the parameters and their values used in all CNN models. Conversely, a brute-force mechanism has been established for the proposed ENL approach to evaluate every conceivable combination of the underlying base learners rigorously. Wherein a combination includes at least two primary learners.

### 4.4. Performance Measure

The evaluation techniques used in this study are based on measures obtained from [62], namely Accuracy (AY), Precision (PN), Recall (RL), F-Measure (FE), Kappa (KA), Log-Loss (LS) and class-specific AUC ROC curves, and Confusion Matrix. These metrics serve as significant benchmarks for assessing the results of the experiment. Accuracy is the ratio of the sum of two accurate predictions (True Positive (TPOS) and True Negative (TNG)) and the total number of data sets (TPOS, TNG, False Positive (FPOS) and False Negative (FNG)) [59]. The accuracy of the model ranges from 1, indicating optimal performance, to 0, indicating minimal effectiveness. The accuracy metric calculates the proportion of accurate predictions for all evaluated instances. The accuracy of a classification model can be determined using (1). Precision is the ratio of positive accurate prediction (TPOS) and summation of two positive predictions (TPOS + FPOS). 1.0 is the best value, and 0.0 is the worst value [59]. The model precision can be calculated using (2). On the contrary, recall is the ratio of positive accurate prediction (TPOS) and the summation of positive accurate prediction (TPOS) and incorrect negative prediction (FNG) [63]. Using Equation (3), we may find out how well a model performs in terms of recall. The weighted mean of recall and precision, based on the weight function β, is called the F score or the F1 score. Using (4) can allow us to calculate the recall of a model. The Kappa coefficient is a metric that contrasts the observed accuracy with the anticipated one. The Kappa coefficient measures classification performance by comparing the test classifier’s performance with that of a random classifier. The metric Kappa can be computed using (5) [64]. The most significant probability-based order unit of measurement is log-loss. The log-loss metric quantifies the uncertainty of a probabilistic approach by evaluating its accuracy in predicting true labels [62]. A low log-loss value suggests an accurate prediction. Using (6) facilitates the computation of a model’s log-loss.

(1) $$\;\mathrm{AY}\;=\frac{TPOS+TNG}{TPOS+TNG+FPOS+FNG}$$

(2) $$\;\mathrm{PN}\;=\frac{TPOS}{TPOS+FPOS}$$

(3) $$\;\mathrm{RL}\;=\frac{TPOS}{TPOS+FNG}$$

(4) $$\;\mathrm{FE}\;=\frac{2\times \;\mathrm{PN}\;\times \;\mathrm{RL}\;}{\;\mathrm{PN}\;+\;\mathrm{RL}\;},$$

(5) $$KA=\frac{\;\mathrm{total}\;\mathrm{accuracy}\;-\;\mathrm{random}\;\mathrm{accuracy}\;}{1-\;\mathrm{random}\;\mathrm{accuracy}\;},$$

(6) $$\;\mathrm{LS}\;=-\frac{1}{N}\sum_{i=1}^{N}d_{i}\cdot \log\left(p\left(d_{i}\right)\right)+\left(1-d_{i}\right)\cdot \log\left(1-p\left(d_{i}\right)\right)$$

where *d* represents the level of the target variable, and p(d) denotes the projected probability of the point reaching the desired value.

The confusion matrix, alternatively referred to as an error matrix, is a tabular representation that depicts an algorithm’s effectiveness, often supervised in nature, within ML and statistical classification domains. The incidences in each true class are represented in the matrix’s rows, and those in each forecasted class are represented in the columns or conversely. The Receiver Operating Characteristic Area Under the Curve (ROC-AUC) metric illustrates the relationship between sensitivity and specificity. It indicates the model’s ability to discriminate [62].

## 5. Results and Discussion

This section presents an examination of the results derived from each model. The pre-trained CNN models have been trained on the imbalance training set in two distinct phases, where all the weights of each layer of the models are kept the same as the original model (Frozen), and second, where all the weights of each layer are trained (Fine-Tuning). Table 3 demonstrates the efficacy of each model on an unbalanced training set. Among all pre-trained models, MobileNetV1 had the best accuracy, precision, recall, and f1-score, 94.32%, 94%, 94%, and 94%, respectively, and Kappa is 90.93%. Then EfficientNetV2B0 comes simultaneously with 93.89% accuracy, 93% precision, recall, and f1-score. The ROC score and log-loss of EfficientNetV2 B0 are 0.990 and 0.303, respectively.

To obtain better performance and to make the evaluation logical and unbiased, the training set has been balanced, and all the models have been applied to the balanced set. Table 4 displays the results of all models on an evenly distributed dataset. In most instances, the overall efficacy of all models has been enhanced. For example, MobileNetV2, NasNetMobile, and EfficientNetV2B0 trained in fine-tune mode indicate the finest accuracy among all pre-trained models.

**Figure 6:**
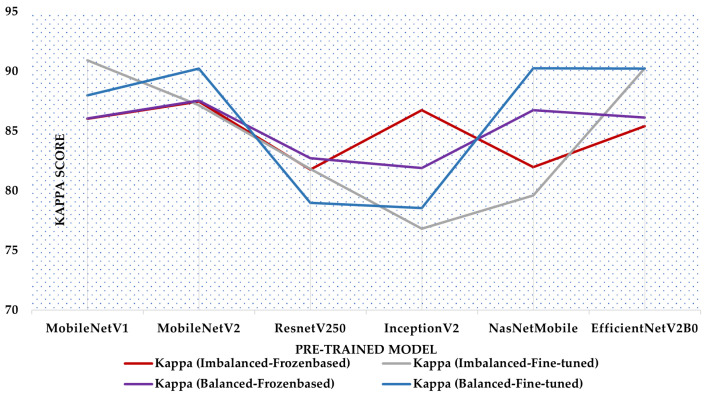
Comparison of Kappa score of different Frozen and Fine-Tune-based transfer learning models prepared from balanced and imbalanced training sets.

The line graph in Figure 6 demonstrates the analogy of the Kappa score of diverse frozen and fine-tune-based transfer learning models prepared from balanced and imbalanced training sets. It is reasonable to observe that the fine-tuned models, namely MobileNetV2, NasNetMobile, and EfficientNetV2B0, trained on a balanced dataset, have demonstrated improved Kappa scores compared to their prior iterations, indicating their higher performance. Frozen-based ResnetV250 prepared from a balanced training set is also responsible for showing the top score compared to its previous states. NasNetMobile has the second-highest accuracy and Kappa score of all pre-trained models. Again, NasNetMobile demonstrates the lowest log-loss, indicating superior probabilistic estimation and uncertainty quantification capabilities. Fine-tune-based MobileNetV1 trained with an imbalanced dataset had the best accuracy and Kappa score of any pre-trained model.

**Figure 7:**
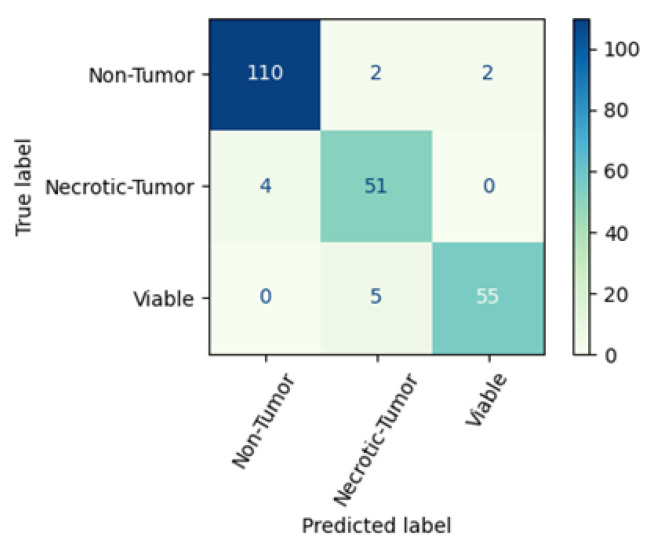
Confusion matrix of MobileNetV1 trained with the imbalanced set.

**Figure 8:**
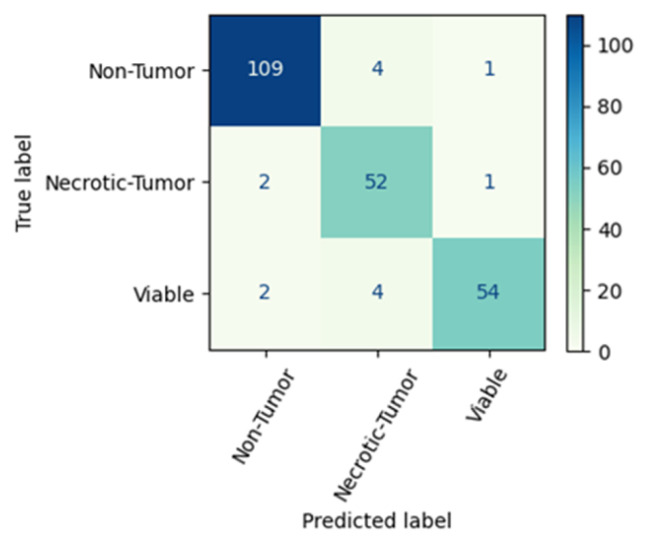
Confusion matrix of NasNetMobile trained with the balanced set.

Confusion matrices in Figure 7 and Figure 8 convey a clear visual of the performance gap between MobileNetV1 and NasNetMobile. The MobileNetV1 model elucidates superior performance in classifying the “Non-Tumor” and “Viable-Tumor” categories. Conversely, the NasNetMobile model accurately classifies instances of the “Necrosis Tumor” class, correctly identifying 52 examples from the test set. These findings underscore the strengths of each model in handling specific tumor classes, providing valuable insights for targeted application and analysis in medical image classification tasks.

The proposed CNN model has also been trained with the same imbalanced training set presented in Table 3. The best results have been obtained from the proposed CNN architecture among all other models prepared from the imbalanced set where the accuracy, precision, recall, f1-score, ROC score, Kappa, and log-loss are 95.20%, 95%, 95%, 95%, 0.995, 92.33%, and 0.129, respectively. In Table 4, it is shown that our proposed CNN architecture has also been trained with a balanced training set. The suggested CNN model’s performance exhibits favorable results compared to current models that have been trained using either a balanced or unbalanced training dataset.The highest accuracy of 95.63% is attained using the suggested CNN approach. Its precision, recall, f1-score, ROC score, Kappa, and log-loss are 95%, 96%, 95%, 0.993, 93.09%, and 0.158, respectively.

**Figure 9:**
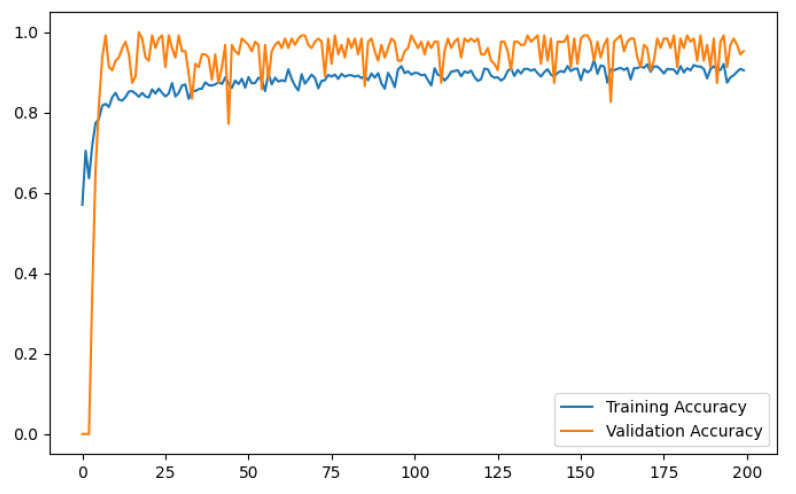
Training and validation accuracy curve of the proposed CNN model.

**Figure 10:**
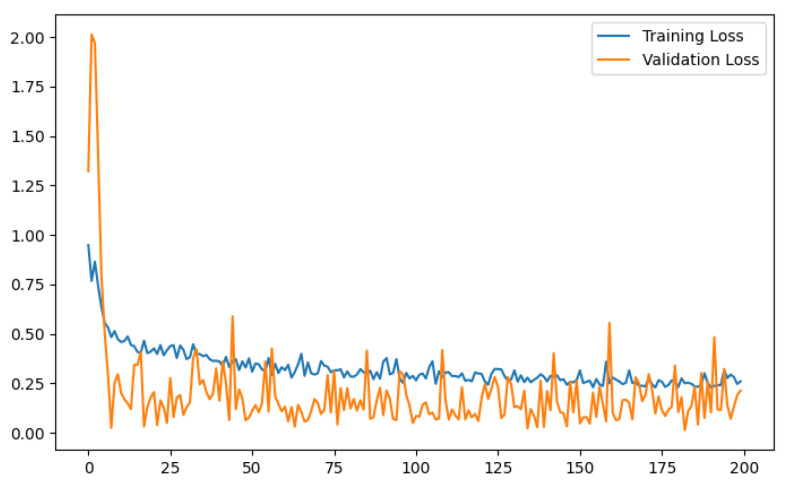
Training and validation loss curve of proposed CNN model.

The training and validation accuracy curves illustrate a gradual increase in the validation accuracy line, closely following the trend of the training accuracy line. Similarly, the training and validation loss curves depict a steady reduction in the validation loss, mirroring the pattern of the training loss. Figure 9 and Figure 10 exhibit graphical representations of the training and validation accuracy and loss curves for the CNN model developed in this study. These figures depict the performance of the model on the balanced dataset. These plots offer valuable insights into the model’s performance and convergence during training, enabling a comprehensive evaluation of its learning capabilities.

**Figure 11:**
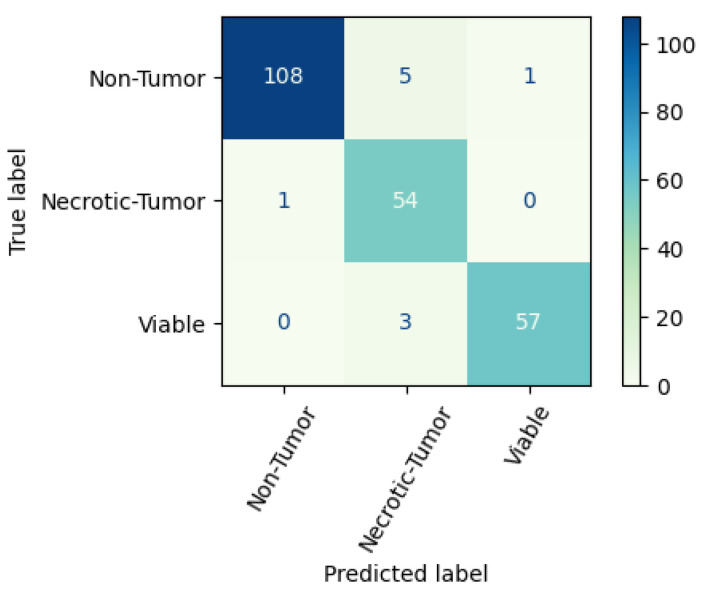
Confusion matrix of the proposed CNN model.

In the test dataset, the number of non-tumor images is 114, whereas the model can classify 108 images correctly. A total of 5 images have been classified as necrotic-tumor and 1 image as viable. In the necrotic-tumor class, the images are 55, whereas 54 images are classified correctly, and 1 image is classified as non-tumor. In the viable class, the total number of images is 60, whereas 57 images are classified correctly, and 3 are classified as necrotic tumors. The confusion matrix of the proposed CNN model on the balanced dataset is shown in Figure 11.

**Figure 12:**
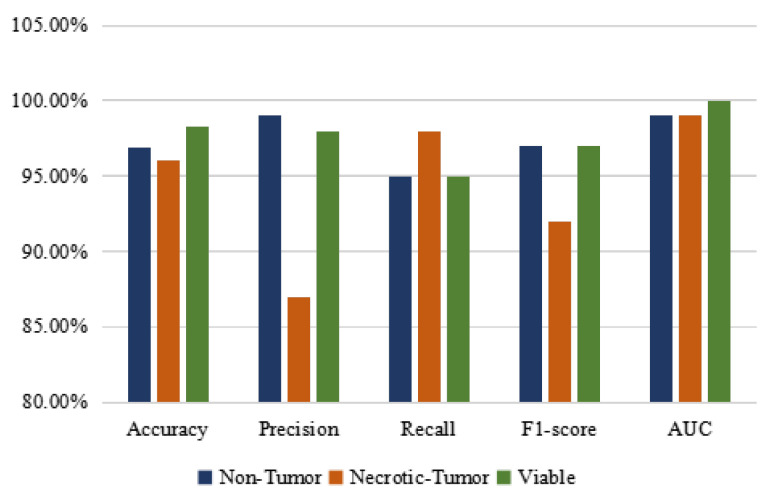
Class-wise accuracy (%), precision (%), recall (%), f1-score (%), and AUC score 603 (%) of the proposed CNN model on a balanced dataset.

Table 5 shows the class-wise performance of the proposed CNN model on a balanced training set. In this context, our proposed CNN model notably achieves the highest levels of accuracy, AUC, and f1-score for the “Viable” class. Additionally, it attains the maximum precision for the “Non-tumor” class and the highest recall for the “Necrotic-Tumor” class. Figure 12 provides a clear comparative visualization of the proposed CNN model’s class-wise accuracy, precision, recall, f1-score, and AUC score on the balanced dataset. The graphical representation allows for an intuitive understanding of the model’s performance across different classes, aiding in assessing its strengths and weaknesses in classifying individual categories.

**Figure 13:**
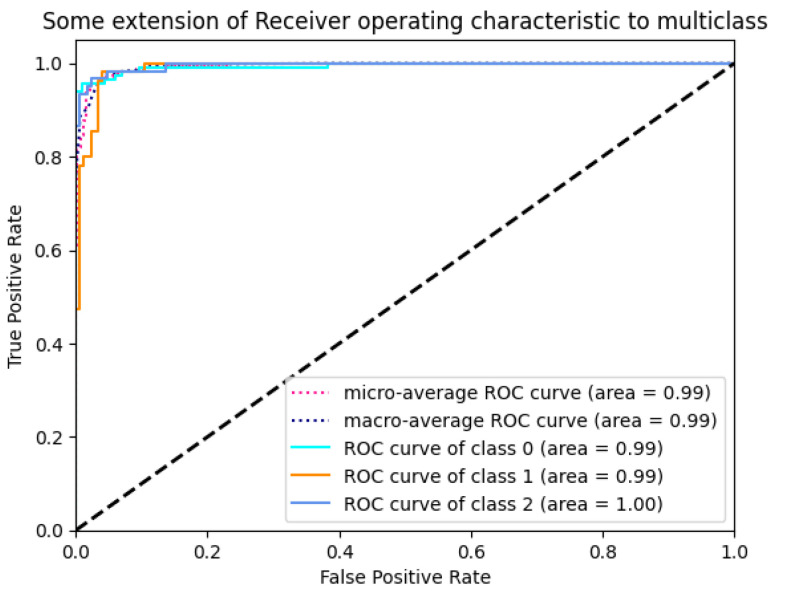
AUC ROC (Receiver Operating Characteristic) curve of proposed CNN model.

In the AUC ROC analysis of the proposed CNN model demonstrated in Figure 13, the micro-average and macro-average AUC achieve an impressive score of 99%.

The findings are obtained from evaluating all combinations of balanced fine-tune-based models, including the suggested CNN model trained on a balanced training set. Wherein a combination includes at least two primary learners. For example, the data contains the performance metrics of three ensemble models that have demonstrated high-performance levels, recorded in Table 6. Table 6 shows that the ensemble model ENL-CNE shows the highest precision, Kappa score, recall, F1 score, and accuracy compared to the other two. ENL-CNE outperforms all other models in terms of accuracy, Kappa score, precision, and F1 score.

Class-wise performance comparison of the proposed CNN model and proposed ensemble learning-based ENL-CNE model has been displayed in Table 7.

**Figure 14:**
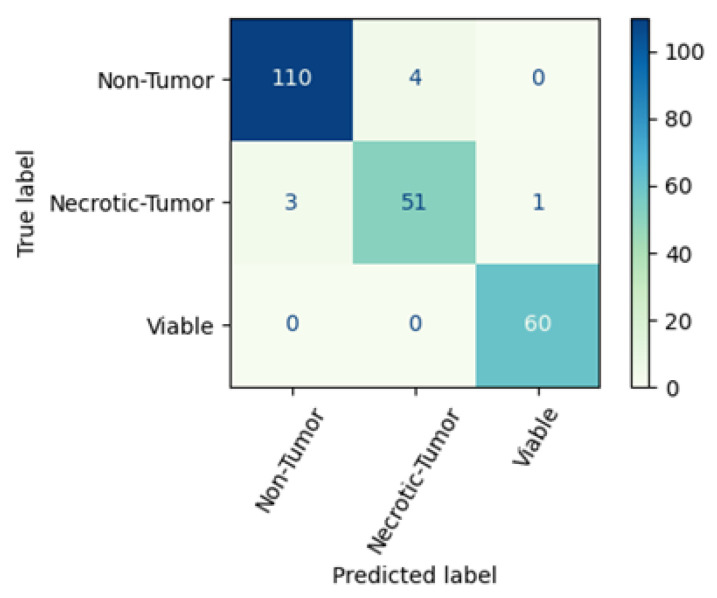
Confusion matrix of proposed ENL-CNE model.

The proposed CNN model has increased precision for non-tumors and superior recall for necrotic tumors. However, the ENL-CNE model outperforms the proposed CNN model in all other circumstances. Figure 14 exhibits the confusion matrix for the proposed ENL-CNE model. One hundred 14 non-tumor images are present within the test set, of which the model accurately classifies 110 images. In the necrotic-tumor class, comprising 55 images, the model correctly classifies 51 images. Similarly, in the viable class, encompassing 60 images, the model achieves precise classification for 60 images. The proposed ENL model achieves an outstanding classification rate for the group of cancerous viable tumors.

The findings of our suggested CNN model are compared in Table 8 with those of other studies that have used the same osteosarcoma dataset. Among existing literature, the analysis performed by Ahmed et al. [26] shows the lowest accuracy from their proposed CNN, and VGG19 is liable for the highest accuracy when Anisuzzaman et al. [31] redacted the analysis. The CNN model introduced by Mishra et al. [32] attained the second-highest accuracy of 92.40% among the existing methodologies. Mahore et al. [13] and Vezakis et al. [28] achieved commendable accuracies of about 91% by employing AdaBoost and MobileNetV2, respectively. Our proposed CNN exceeded these figures with an accuracy of 95.63%. Furthermore, our novel approach, the proposed ENL-CNE classifier, which is an ENL-based model composed of the suggested CNN, fine-tuned NasNetMobile, and EfficientNetV2B0 base learners, pushed the boundaries even further, achieving an impressive accuracy of 96.51%. The comparative analysis underscores the robustness of our methodologies and their potential to advance the field’s standard of exactness. Even though different validation methods affect comparisons, our study’s success shines and shows how far our research has come in accuracy.

**Figure 15:**
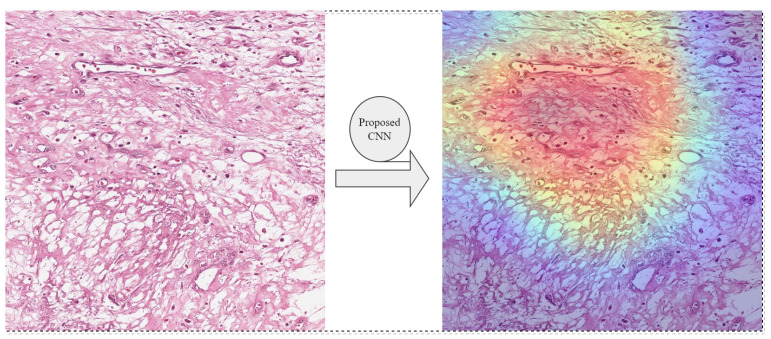
Proposed CNN model’s interpretability using Grad-CAM technique for Necrotic-Tumor category.

The Gradient-weighted Class Activation Mapping (Grad-CAM) technique has been employed to enhance the interpretability of our model’s visualization. The CNN modules are designed to extract information from images at multiple layers, therefore capturing a range of levels of abstraction. The Grad-CAM technique utilizes the gradients of the score of the target class to the feature maps of a specific convolutional layer. These gradients indicate how changes in the feature maps affect the final classification score [65]. In Figure 15, Grad-CAM provides a visualization that helps to interpret and understand our proposed CNN’s decision-making process, making it more transparent and explainable.

## 6. Conclusions

This study presents a novel CNN architecture and an adapted heterogeneous ensemble learning-based voting classifier prepared from proposed CNN, fine-tuned NasNetMobile, and fine-tuned Efficient-NetV2B0 base learners to classify osteosarcoma effectively. Due to intra-class changes, inter-class similarities, crowded con-texts, and inconsistent data, the classification and prediction of a limited dataset with CNN architecture are challenging and complex. As imbalanced data negatively affects model performance and is responsible for the model’s unbiasedness, a balanced training set was developed using an image augmentation technique to counteract these obstacles. Subsequently, the proposed CNN model and adapted heterogeneous ensemble learning-based voting classifier have been developed to classify the tumor, non-tumor, and necrotic tumor cells. In addition, six pre-trained CNN models were also trained in frozen and fine-tuned cases. However, our proposed CNN model functions well on the balanced dataset and outperforms all pre-trained models. However, our proposed CNN and adapted heterogeneous ensemble learning-based voting classifier unequivocally outperform all competing models. Hence, the equivalent CNN architecture and proposed voting classifier can be applied to different forms of cancer, enabling the creation of a generic model capable of analyzing diverse histology datasets for medical diagnosis. The findings of this study have practical implications in telemedicine, mobile healthcare systems, and as a supportive tool for medical professionals. Our research will also continue investigating different neural network training topologies and strategies for categorizing various medical photos and identifying tumors. 

## Figures and Tables

**Table 1:** Detail description of proposed CNN layers.

Layer	Filter Size (f)	Depth	Input Shape (IS)	Output Shape (OS)	Parameters
Batch Normalization	-	-	224 × 224 × 3	224 × 224 × 3	12
Conv2D layer 1	3 × 3	32	224 × 224 × 3	224 × 224 × 32	896
Pooling layer 1	2 × 2	32	224 × 224 × 32	112 × 112 × 32	0
Conv2D layer 2	3 × 3	32	112 × 112 × 32	112 × 112 × 32	9248
Pooling layer 2	2 × 2	32	112 × 112 × 32	56 × 56 × 32	0
Conv2D layer 3	3 × 3	64	56 × 56 × 32	56 × 56 × 64	18,496
Pooling layer 3	2 × 2	64	56 × 56 × 64	28 × 28 × 64	0
Conv2D layer 4	3 × 3	64	28 × 28 × 64	28 × 28 × 64	36,928
Pooling layer 4	2 × 2	64	28 × 28 × 64	14 × 14 × 64	0
Conv2D layer 5	3 × 3	128	14 × 14 × 64	14 × 14 × 128	73,856
Pooling layer 5	2 × 2	128	14 × 14 × 128	7 × 7 × 128	0
Conv2D layer 6	3 × 3	128	7 × 7 × 128	7 × 7 × 128	147,584
Pooling layer 6	2 × 2	128	7 × 7 × 128	3 × 3 × 128	0
Conv2D layer 7	3 × 3	256	3 × 3 × 128	3 × 3 × 256	295,168
Pooling layer 7	2 × 2	256	3 × 3 × 256	1 × 1 × 256	0
Flatten layer		256	1 × 1 × 256	256	0
Dense layer 1		512	256	512	131,584
Dropout layer 1		512	512	512	0
Dense layer 2		3	512	3	1539
(SoftMax)					
Total Parameters					715,311

**Table 2:** Chosen parameters value for all CNN models.

Parameter Name	Value
optimizer	Adam
loss-function	Sparse Categorical cross-entropy
learning_rate	0.001
batch_size	16
epochs	200

**Table 3:** Accuracy (%), Precision (%), Recall (%), F1-Score (%), AUC, Kappa (%), and Log-loss of all models on an imbalanced training set.

Phase	Algorithm	Accuracy	Precision	Recall	F1-Score	AUC	Kappa	Log-Loss
Froze-based	MobileNetV1	91.27	90	90	90	0.981	86.02	0.519
	MobileNetV2	92.14	91	91	91	0.986	87.46	0.407
	ResnetV250	88.65	88	87	88	0.981	81.77	0.437
	InceptionV2	91.7	91	91	91	0.984	86.74	0.375
	NasNetMobile	88.65	87	88	88	0.973	81.98	0.54
	EfficientNetV2B0	90.83	89	90	90	0.98	85.4	0.353
Fine-tuned	MobileNetV1	94.32	94	94	94	0.975	90.93	0.474
	MobileNetV2	92.14	93	90	91	0.983	87.17	0.395
	ResnetV250	88.65	88	88	88	0.975	81.81	0.381
	InceptionV2	85.15	84	86	84	0.958	76.83	1.06
	NasNetMobile	87.77	92	84	86	0.971	79.6	0.736
	EfficientNetV2B0	93.89	93	93	93	0.99	90.25	0.303
Complete Training	CNN	95.2	95	95	95	0.995	92.33	0.129

**Table 4:** Accuracy (%), Precision (%), Recall (%), F1-Score (%), AUC, Kappa (%), and Log-loss of all models on a balanced training set.

Phase	Algorithm	Accuracy	Precision	Recall	F1-Score	AUC	Kappa	Log-Loss
Frozen-based	MobileNetV1	91.27	90	90	90	0.982	86.04	0.62
	MobileNetV2	92.14	91	92	92	0.981	87.55	0.582
	ResnetV250	89.08	88	89	88	0.98	82.72	0.565
	InceptionV2	88.65	88	88	87	0.977	81.91	0.51
	NasNetMobile	91.7	91	91	91	0.981	86.75	0.459
	EfficientNetV2B0	91.27	90	90	90	0.978	86.12	0.461
Fine-tuned	MobileNetV1	92.58	93	91	92	0.989	87.97	0.248
	MobileNetV2	93.89	93	93	93	0.989	90.22	0.237
	ResnetV250	86.9	86	85	86	0.965	78.99	0.432
	InceptionV2	86.9	88	83	85	0.979	78.55	0.496
	NasNetMobile	93.89	93	93	93	0.991	90.26	0.222
	EfficientNetV2B0	93.89	93	93	93	0.991	90.22	0.23
Complete Training	CNN	95.63	95	96	95	0.993	93.09	0.158

**Table 5:** Class-wise Accuracy (%), Precision (%), Recall (%), F1-Score (%), AUC of the proposed CNN model on Balanced Dataset.

Classes	Accuracy	Precision	Recall	F1-Score	AUC
Non-Tumor	96.94	99	95	97	0.99
Necrotic-Tumor	96.07	87	98	92	0.99
Viable	98.25	98	95	97	1

**Table 6:** Performance comparison of three highly performing ensemble learning models generated from the idea of brute-force approach.

EL	Models	Accuracy	Precision	Recall	F1-Score	Kappa
ENL-MNE	MobileNetV2, NasNetMobile, EfficientNetV2B0	92.14	0.9236	0.9214	0.922	87.55
ENL-CMINE	CNN, MobileNetV2, InceptionV2, NasNetMobile, EfficientNetV2B0	94.32	0.9437	0.9432	0.9434	94.32
ENL-CNE	CNN, NasNetMobile, EfficientNetV2B0	96.51	0.965	0.9651	0.965	96.5

**Table 7:** Comparison of class-wise precision (%), Recall (%), and F1-Score (%) of the proposed CNN model and ENL-CNE model on the balanced training set.

	Proposed CNN			Proposed ENL-CNE		
**Classes**	**Precision**	**Recall**	**F1-Score**	**Precision**	**Recall**	**F1-Score**
Non-Tumor	99	95	97	97	96	97
Necrotic-Tumor	87	98	92	93	93	93
Viable	98	95	97	98	100	99

**Table 8:** Comparative evaluation of exactness with other investigations.

Study	Approach	Validation Method	Overall Accuracy (%)
Mahore et al. [13]	Adaboost	Holdout	91.7
Ahmed et al. [26]	CNN	Holdout	86
Gawade et al. [27]	ResNet101	Holdout	90.36
Vezakis et al. [28]	MobileNetV2	Cross-Validation	91
Nabid et al. [30]	Sequential RCNN	Holdout	89
Anisuzzaman et al. [31]	VGG19	Holdout	93.91
Mishra et al. [32]	CNN	Holdout	92.4
**Our Study**	**Proposed CNN**	**Holdout**	**95.63**
	**Proposed ENL-CNE**	**Holdout**	**96.51**

## Data Availability

The datasets utilized in this article were obtained from “Osteosarcoma data from UT Southwest-ern/UT Dallas for Viable and Necrotic-Tumor Assessment (Osteosarcoma Tumor Assessment)” webpage, which is freely accessible for all scientists and investigators to conduct experiments and can be accessed through website: https://wiki.cancerimagingarchive.net/pages/viewpage.action?pageId=52756935 (accessed on 4 October 2023).

## References

[1] Biermann J.S., Adkins D.R., Agulnik M., Benjamin R.S., Brigman B., Butrynski J.E., Cheong D., Chow W., Curry W.T., Frassica D.A. (2013). Bone Cancer. J. Natl. Compr. Cancer Netw..

[2] Jimenez-Andrade J.M., Mantyh W.G., Bloom A.P., Ferng A.S., Geffre C.P., Mantyh P.W. (2010). Bone cancer pain. Ann. N. Y. Acad. Sci..

[3] Ottaviani G., Jaffe N. (2009). Pediatric and Adolescent Osteosarcoma.

[4] Ritter J., Bielack S. (2010). Osteosarcoma. Ann. Oncol..

[5] Ozaki T., Flege S., Liljenqvist U., Hillmann A., Delling G., Salzer-Kuntschik M., Jürgens H., Kotz R., Winkelmann W., Bielack S.S. (2002). Osteosarcoma of the spine. Cancer.

[6] Jafari F., Javdansirat S., Sanaie S., Naseri A., Shamekh A., Rostamzadeh D., Dolati S. (2020). Osteosarcoma: A comprehensive review of management and treatment strategies. Ann. Diagn. Pathol..

[7] Litjens G., Sánchez C.I., Timofeeva N., Hermsen M., Nagtegaal I., Kovacs I., van de Kaa C.H., Bult P., van Ginneken B., van der Laak J. (2016). Deep learning as a tool for increased accuracy and efficiency of histopathological diagnosis. Sci. Rep..

[8] Meggendorfer M., Jobanputra V., Wrzeszczynski K.O., Roepman P., de Bruijn E., Cuppen E., Buttner R., Caldas C., Grimmond S., Mullighan C.G. (2022). Analytical demands to use whole-genome sequencing in precision oncology. Semin. Cancer Biol..

[9] Ben-Cohen A., Greenspan H. (2020). Liver Lesion Detection in CT Using Deep Learning Techniques.

[10] Aljuaid H., Alturki N., Alsubaie N., Cavallaro L., Liotta A. (2022). Computer-aided diagnosis for breast cancer classification using deep neural networks and transfer learning. Comput. Methods Programs Biomed..

[11] Fu Y., Xue P., Ji H., Cui W., Dong E. (2020). Deep model with Siamese network for viable and necrotic tumor regions assessment in osteosarcoma. Med. Phys..

[12] Asmaria T., Mayasari D.A., Heryanto M.A., Kurniatie M., Wati R., Aurellia S. (2021). Osteosarcoma Classification Using Convolutional Neural Network.

[13] Mahore S., Bhole K., Rathod S. Comparative Analysis of Machine Learning Algorithm for Classification of different Osteosarcoma types. Proceedings of the 12th International Conference on Computing Communication and Networking Technologies (ICCCNT).

[14] Xue D., Zhou X., Li C., Yao Y., Rahaman M.M., Zhang J., Chen H., Zhang J., Qi S., Sun H. (2020). An Application of Transfer Learning and Ensemble Learning Techniques for Cervical Histopathology Image Classification. IEEE Access.

[15] Saxena U., Moulik S., Nayak S.R., Hanne T., Roy D.S. (2021). Ensemble-Based Machine Learning for Predicting Sudden Human Fall Using Health Data. Math. Probl. Eng..

[16] Leavey P., Sengupta A., Rakheja D., Daescu O., Arunachalam H., Mishra R. (2019). Osteosarcoma Data from UT Southwestern/UT Dallas for Viable and Necrotic Tumor Assessment [Data Set]. https://wiki.cancerimagingarchive.net/pages/viewpage.action?pageId=52756935.

[17] Walid M.A.A., Ahmed S.M., Zeyad M., Galib S.M.S., Nesa M. (2022). Analysis of machine learning strategies for prediction of passing undergraduate admission test. Int. J. Inf. Manag. Data Insights.

[18] Breeden J.L., Leonova E. (2021). Creating Unbiased Machine Learning Models by Design. J. Risk Financ. Manag..

[19] Bi C., Wang J., Duan Y., Fu B., Kang J.R., Shi Y. (2022). MobileNet Based Apple Leaf Diseases Identification. Mob. Netw. Appl..

[20] Buiu C., Dănăilă V.R., Răduţă C.N. (2020). MobileNetV2 Ensemble for Cervical Precancerous Lesions Classification. Processes.

[21] Pedersen M., Andersen M.B., Christiansen H., Azawi N.H. (2020). Classification of renal tumour using convolutional neural networks to detect oncocytoma. Eur. J. Radiol..

[22] Halawa L.J., Wibowo A., Ernawan F. Face Recognition Using Faster R-CNN with Inception-V2 Architecture for CCTV Camera. Proceedings of the 2019 3rd International Conference on Informatics and Computational Sciences (ICICoS).

[23] Cakmak M., Tenekeci M.E. Melanoma detection from dermoscopy images using Nasnet Mobile with Transfer Learning. Proceedings of the 2021 29th Signal Processing and Communications Applications Conference (SIU).

[24] Venkatesh, Sheela R.K., Nagaraju Y., Sahu D.A. Histopathological Image Classification of Breast Cancer using EfficientNet. Proceedings of the 2022 3rd International Conference for Emerging Technology (INCET).

[25] Spanhol F.A., Oliveira L.S., Petitjean C., Heutte L. Breast cancer histopathological image classification using Convolutional Neural Networks. Proceedings of the 2016 International Joint Conference on Neural Networks (IJCNN).

[26] Ahmed I., Sardar H., Aljuaid H., Khan F.A., Nawaz M., Awais A. (2021). Convolutional Neural Network for Histopathological Osteosarcoma Image Classification. Comput. Mater. Contin..

[27] Gawade S., Bhansali A., Patil K., Shaikh D. (2023). Application of the convolutional neural networks and supervised deep-learning methods for osteosarcoma bone cancer detection. Healthc. Anal..

[28] Vezakis I.A., Lambrou G.I., Matsopoulos G.K. (2023). Deep Learning Approaches to Osteosarcoma Diagnosis and Classification: A Comparative Methodological Approach. Cancers.

[29] Shen R., Li Z., Zhang L., Hua Y., Mao M., Li Z., Cai Z., Qiu Y., Gryak J., Najarian K. Osteosarcoma Patients Classification Using Plain X-Rays and Metabolomic Data. Proceedings of the 2018 40th Annual International Conference of the IEEE Engineering in Medicine and Biology Society (EMBC).

[30] Nabid R.A., Rahman M.L., Hossain M.F. Classification of Osteosarcoma Tumor from Histological Image Using Sequential RCNN. Proceedings of the 2020 11th International Conference on Electrical and Computer Engineering (ICECE).

[31] Anisuzzaman D., Barzekar H., Tong L., Luo J., Yu Z. (2021). A deep learning study on osteosarcoma detection from histological images. Biomed. Signal Process. Control..

[32] Mishra R., Daescu O., Leavey P., Rakheja D., Sengupta A. (2018). Convolutional Neural Network for Histopathological Analysis of Osteosarcoma. J. Comput. Biol..

[33] Ho X.D., Nguyen H.G., Trinh L.H., Reimann E., Prans E., Kõks G., Maasalu K., Le V.Q., Nguyen V.H., Le N.T. (2017). Analysis of the expression of repetitive DNA elements in osteosarcoma. Front. Genet..

[34] Reimann E., Kõks S., Ho X.D., Maasalu K., Märtson A. (2014). Whole exome sequencing of a single osteosarcoma case—integrative analysis with whole transcriptome RNA-seq data. Hum. Genom..

[35] Asito L.Y., Pereira H.M., Nogueira-Barbosa M.H., Tinós R. Detection of osteosarcoma on bone radiographs using convolutional neural networks. Proceedings of the Anais do 15. Congresso Brasileiro de Inteligência Computacional.

[36] Pham K., Kim D., Park S., Choi H. (2021). Ensemble learning-based classification models for slope stability analysis. CATENA.

[37] Nalini T., Rama A. (2022). Impact of temperature condition in crop disease analyzing using machine learning algorithm. Meas. Sens..

[38] Waibel A., Hanazawa T., Hinton G., Shikano K., Lang K. (1989). Phoneme recognition using time-delay neural networks. IEEE Trans. Acoust. Speech Signal Process..

[39] Lecun Y., Bottou L., Bengio Y., Haffner P. (1998). Gradient-based learning applied to document recognition. Proc. IEEE.

[40] Alzubaidi L., Zhang J., Humaidi A.J., Al-Dujaili A., Duan Y., Al-Shamma O., Santamaría J., Fadhel M.A., Al-Amidie M., Farhan L. (2021). Review of deep learning: Concepts, CNN architectures, challenges, applications, future directions. J. Big Data.

[41] Minaee S., Boykov Y.Y., Porikli F., Plaza A.J., Kehtarnavaz N., Terzopoulos D. (2021). Image Segmentation Using Deep Learning: A Survey. IEEE Trans. Pattern Anal. Mach. Intell..

[42] Zhang Y.D., Pan C., Sun J., Tang C. (2018). Multiple sclerosis identification by convolutional neural network with dropout and parametric ReLU. J. Comput. Sci..

[43] Zhang C.L., Luo J.H., Wei X.S., Wu J. (2017). In Defense of Fully Connected Layers in Visual Representation Transfer. Advances in Multimedia Information Processing—PCM 2017.

[44] Rabano S.L., Cabatuan M.K., Sybingco E., Dadios E.P., Calilung E.J. Common Garbage Classification Using MobileNet. Proceedings of the 2018 IEEE 10th International Conference on Humanoid, Nanotechnology, Information Technology, Communication and Control, Environment and Management (HNICEM).

[45] Sae-Lim W., Wettayaprasit W., Aiyarak P. Convolutional Neural Networks Using MobileNet for Skin Lesion Classification. Proceedings of the 2019 16th International Joint Conference on Computer Science and Software Engineering (JCSSE).

[46] Patel R., Chaware A. Transfer Learning with Fine-Tuned MobileNetV2 for Diabetic Retinopathy. Proceedings of the 2020 International Conference for Emerging Technology (INCET).

[47] Dong K., Zhou C., Ruan Y., Li Y. MobileNetV2 Model for Image Classification. Proceedings of the 2020 2nd International Conference on Information Technology and Computer Application (ITCA).

[48] He K., Zhang X., Ren S., Sun J. Deep Residual Learning for Image Recognition. Proceedings of the 2016 IEEE Conference on Computer Vision and Pattern Recognition (CVPR).

[49] Sarwinda D., Paradisa R.H., Bustamam A., Anggia P. (2021). Deep Learning in Image Classification using Residual Network (ResNet) Variants for Detection of Colorectal Cancer. Procedia Comput. Sci..

[50] Mustafa T., Dhavale S., Kuber M.M. (2020). Performance Analysis of Inception-v2 and Yolov3-Based Human Activity Recognition in Videos. SN Comput. Sci..

[51] Addagarla S.K. (2020). Real Time Multi-Scale Facial Mask Detection and Classification Using Deep Transfer Learning Techniques. Int. J. Adv. Trends Comput. Sci. Eng..

[52] Deng L., Suo H., Li D. (2022). Deepfake Video Detection Based on EfficientNet-V2 Network. Comput. Intell. Neurosci..

[53] Marques G., Agarwal D., de la Torre Díez I. (2020). Automated medical diagnosis of COVID-19 through EfficientNet convolutional neural network. Appl. Soft Comput..

[54] Tan M., Le Q.V. (2021). EfficientNetV2: Smaller Models and Faster Training. arXiv.

[55] Yu Z., Haghighat F., Fung B.C., Yoshino H. (2010). A decision tree method for building energy demand modeling. Energy Build..

[56] Barus O.P., Happy J., Jusin, Pangaribuan J.J., H S.Z., Nadjar F. Liver Disease Prediction Using Support Vector Machine and Logistic Regression Model with Combination of PCA and SMOTE. Proceedings of the 2022 1st International Conference on Technology Innovation and Its Applications (ICTIIA).

[57] Beuque M., Martin-Lorenzo M., Balluff B., Woodruff H.C., Lucas M., de Bruin D.M., van Timmeren J.E., Boer O.J., Heeren R.M., Meijer S.L. (2021). Machine learning for grading and prognosis of esophageal dysplasia using mass spectrometry and histological imaging. Comput. Biol. Med..

[58] Xu Y., Lam H.K., Jia G. (2021). MANet: A two-stage deep learning method for classification of COVID-19 from Chest X-ray images. Neurocomputing.

[59] Sharmili K.C., Suja G.P., Pandian E., Walid M.A.A., Arunachalam S., Babu G. An Effective Diagnosis of Alzheimer’s Disease with the Use of Deep Learning based CNN Model. Proceedings of the 2023 7th International Conference on Intelligent Computing and Control Systems (ICICCS).

[60] Yadav S.S., Jadhav S.M. (2019). Deep convolutional neural network based medical image classification for disease diagnosis. J. Big Data.

[61] Bechelli S., Delhommelle J. (2022). Machine Learning and Deep Learning Algorithms for Skin Cancer Classification from Dermoscopic Images. Bioengineering.

[62] Uddin M.J., Ahamad M.M., Hoque M.N., Walid M.A.A., Aktar S., Alotaibi N., Alyami S.A., Kabir M.A., Moni M.A. (2023). A Comparison of Machine Learning Techniques for the Detection of Type-2 Diabetes Mellitus: Experiences from Bangladesh. Information.

[63] Walid M.A.A., Ahmed S.M., Sadique S.M.S. A Comparative Analysis of Machine Learning Models for Prediction of Passing Bachelor Admission Test in Life-Science Faculty of a Public University in Bangladesh. Proceedings of the 2020 IEEE Electric Power and Energy Conference (EPEC).

[64] Hassan M.M., Mollick S., Yasmin F. (2022). An unsupervised cluster-based feature grouping model for early diabetes detection. Healthc. Anal..

[65] Selvaraju R.R., Cogswell M., Das A., Vedantam R., Parikh D., Batra D. Grad-CAM: Visual Explanations from Deep Networks via Gradient-Based Localization. Proceedings of the 2017 IEEE International Conference on Computer Vision (ICCV).

